# The Myotonic Plot Thickens: Electrical Myotonia in Antimuscle-Specific Kinase Myasthenia Gravis

**DOI:** 10.1155/2015/242691

**Published:** 2015-12-03

**Authors:** Marcus Magnussen, Ioannis Karakis, Taylor B. Harrison

**Affiliations:** Department of Neurology, Emory University, Atlanta, GA 30329, USA

## Abstract

Electrical myotonia is known to occur in a number of inherited and acquired disorders including myotonic dystrophies, channelopathies, and metabolic, toxic, and inflammatory myopathies. Yet, electrical myotonia in myasthenia gravis associated with antibodies against muscle-specific tyrosine kinase (MuSK) has not been previously reported. We describe two such patients, both of whom had a typical presentation of proximal muscle weakness with respiratory failure in the context of a significant electrodecrement in repetitive nerve stimulation. In both cases, concentric needle examination revealed electrical myotonia combined with myopathic motor unit morphology and early recruitment. These findings suggest that MuSK myasthenia should be included within the differential diagnosis of disorders with electrical myotonia.

## 1. Introduction

Muscle-specific tyrosine kinase myasthenia gravis (MuSK-MG) is a type of myasthenia that is characterized by bulbar-predominant symptoms that tend to be refractory to pyridostigmine therapy. Nearly 10–20% of MG patients are seronegative for Acetylcholine Receptor Antibody (AChR-Ab), and several studies have found that 20–40% of these AChR-Ab negative patients in the United States test positive for MuSK Ab [1–3]. Worldwide, the number of AChR-Ab negative patients who are MuSK Ab positive is estimated to be near 40–60% [1–3].

MuSK-MG may occasionally mimic myopathy both on clinical and electrophysiological grounds. Clinically, atrophy of bulbar and proximal muscle groups has commonly been described [1, 4]. Electrophysiologically, a myopathic pattern has also been reported during needle EMG testing in MuSK-MG patients, at times with muscle membrane irritability in the form of fibrillation potentials and positive sharp waves [4, 5]. Electrical myotonia in cases of MuSK-MG, however, is so far unrecognized. Herein, we report two such cases and attempt to provide plausible explanations for its occurrence along with practical ramifications.

## 2. Case Presentations

### 2.1. Case  1

A 45-year-old African American female presented with complaints of progressive generalized weakness, weight loss, fatigue, and dyspnea of 8-month duration. Her symptoms began with diarrhea, weight loss, and fatigue. Her diarrhea resolved within weeks, but she continued to suffer from dyspnea on exertion which eventually persisted at rest. At presentation, she was also complaining of proximal upper extremity weakness, generalized fatigue, and mild dysphagia. She denied diplopia, ptosis, rash, or arthralgias. There was no history of statin or other myotoxic medication use. There was no family history of neurological illness or consanguinity. She demonstrated 4/5 nonfatigable weakness in proximal shoulder and hip girdle musculature, as well as in the neck flexors and extensors, based on the Medical Research Council (MRC) scale. Strength testing of the distal upper and lower extremities was full, and there were no clinical signs of myotonia. Her cranial nerve exam was significant for subtle bifacial weakness. The remainder of her exam revealed normal sensation, reflexes, and coordination testing.

She was admitted to the intensive care unit due to concern for worsening respiratory failure. Spirometry showed a reduced forced vital capacity that was 81% of the predicted value. Laboratory testing revealed a respiratory acidosis, hypercapnia, and a compensatory metabolic alkalosis.

Routine nerve conduction studies (NCSs) showed no significant abnormalities. Electromyography (EMG) of selected proximal and distal muscles in the right upper and lower extremities showed small amplitude and polyphasic motor units action potentials (MUAPs) with early recruitment in tibialis anterior and deltoid. Iliopsoas showed normal MUAP morphology with early recruitment. Myotonic discharges were observed in each of these muscles. Vastus lateralis, medial gastrocnemius, and triceps testing were normal. Thoracic paraspinal muscles showed moderate fibrillations and positive waves with small amplitude, polyphasic MUAPs demonstrating a normal recruitment pattern.

Creatine kinase (CK), thyroid stimulating hormone, and leukocyte acid *α*-glucosidase activity were normal. Genetic testing for myotonic dystrophy (DM2) showed 134 CCTG repeats, within normal limits. Subsequent muscle biopsy showed nonspecific mild type 2 fiber atrophy without evidence for myopathy. Repetitive nerve stimulation (RNS) at 3 Hz revealed >10% decrement when stimulating the right spinal accessory and right facial nerves. Serum AChR-Ab (including binding, modulating, and striational antibodies) were negative. Serum MuSK Ab testing (via radioimmunoassay (RIA) using highly purified MuSK antigen) was positive with a titer in excess of 10240 Units, leading to the diagnosis of MuSK-MG. Chest CT showed no evidence for thymoma. The patient was initially treated with intravenous immunoglobulin, azathioprine, and steroids. She was readmitted with a myasthenia exacerbation and received plasmapheresis (PLEX). Following PLEX she remained well controlled on azathioprine with continued functional improvement.

### 2.2. Case  2

A 54-year-old female presented with approximately one decade of proximal, painless, symmetric upper and lower extremity and neck flexor weakness. There was concomitant fluctuating respiratory insufficiency requiring periodic intubations as well as home use of oxygen and biphasic noninvasive ventilation. The patient denied diplopia, ptosis, dysarthria, or dysphagia. She denied rash, rheumatological complaints, or systemic complaints. She had no history of statin or other myotoxic medication use. There was no definite neurological family history or history of consanguinity. Neurology was consulted regarding suspicion for neuromuscular respiratory failure.

Initial EDX testing showed normal sensory and motor NCSs and F responses. Concentric needle EMG suggested a myopathy with myotonic discharges seen in the cervical paraspinal and iliopsoas muscles. Serum CK and TSH were both within the range of normal. AChR-Ab, voltage-gated calcium channel antibodies, and a rheumatological panel were negative. A Tensilon test did not lead to any remarkable clinical improvement. An echocardiogram showed moderate concentric hypertrophy with preserved ejection fraction. Spirometry identified a restrictive pattern. A diaphragmatic study showed weak but preserved motility of both hemidiaphragms. CT of the chest showed emphysematous changes and was otherwise unremarkable. Her exam during an admission that required intubation revealed mild, fluctuating restriction in conjugate gaze to the right without ptosis, diplopia, and normal pupillary responses. Lower facial musculature and lingual strength was hard to fully appreciate due to intubation but appeared mildly-to-moderately weak without overt atrophy. Neck flexors were 4−/5 and bilateral deltoids and triceps 4+/5 on the MRC scale. Bilateral hip flexors were 5−/5 and more distal lower extremity muscles strength was full. There was mild-to-moderate shoulder girdle and periscapular atrophy. No fasciculations were evident. There was no clinical myotonia. More distal upper extremity muscles were full. Deep tendon reflexes and sensory and cerebellar examination were normal.

A repeat EDX study revealed a decrement when stimulating the facial nerve and redemonstrated electrical myotonia during needle exam of proximal muscle groups only (iliopsoas and deltoid). White blood cell *α*-glucosidase activity was normal. MuSK antibody testing via RIA yielded highly abnormal titers in excess of 10240 Units. The patient was treated with PLEX and a regimen of azathioprine and steroids was initiated with dramatic improvement in her facial, limb girdle, and respiratory muscle weakness. Ultimately, she was able to wean from the ventilator and has continued to experience improved functionality.

## 3. Discussion

These cases both involve African American women with proximal muscle weakness and respiratory insufficiency that first began in the 5th decade of life. Diagnostic testing was significant for markedly elevated serum MuSK antibody titers as well as 3 Hz RNS leading to significant (>10%) electrodecrement, leading to a diagnosis of MuSK-MG. Uniquely, both patients also exhibited findings suggestive of an irritable myopathy with prominent electrical myotonia on needle EMG. Both patients experienced clinical improvement following a course of PLEX and initiation of azathioprine therapy.

The clinical presentation of MuSK-MG is variable. A portion of these patients will have predominant weakness of the neck and bulbar and respiratory musculature, with or without muscle atrophy [1, 6–8]. A separate subset of these patients will also present with isolated ocular paresis, while others will undergo a clinical course that is indistinguishable from AChR-Ab+ MG [1]. Clinical features that may distinguish MUSK-MG patients from AChR-Ab MG include muscle atrophy, bulbar-predominant symptoms and signs, and poor response to pyridostigmine therapy. It is not clear at this time whether MUSK-MG displays a unique response to immunomodulatory therapy, though studies have suggested a less robust response to IVIG as compared to AChR-Ab+ MG.

MuSK-MG may have RNS abnormalities limited to the facial and proximal (e.g., trapezius) muscles, more focal abnormalities with SFEMG, and myopathic motor unit action potentials on concentric needle exam [1, 6–8]. These findings are contrasted by those with classical generalized MG, which typically reveal more diffuse RNS and SFEMG abnormalities without myopathic morphology observed on EMG. Myopathic motor unit morphology, with an early recruitment pattern during concentric needle electromyography, has been previously reported with MG [4, 5]. The proposed mechanism for the observation of myopathic features in MG has been myofibrillar necrosis or the presence of impulse blocking within the motor unit, which may shorten the amplitude and duration of the motor unit action potential [9]. Loss of synchronous firing of muscle fibers within a particular motor unit may contribute to the observed polyphasia.

The observed electrophysiological abnormalities in AChR+ and MuSK-MG likely reflect differences between AChR+ and MuSK-MG pathogenesis described at the molecular and histopathological levels. In AChR+ MG, compliment-mediated damage at the level of the postsynaptic membrane results in a reduction of AChRs and simplification of postsynaptic folds, representing a major mechanism for the disease process. In MuSK-MG, animal models of experimental autoimmune myasthenia gravis have demonstrated both pre- and postsynaptic neuromuscular transmission defects [10, 11]. MuSK-MG is additionally associated with the IgG4 antibody class, which does not activate compliment, and antibody titer has been correlated with clinical severity [12]. While MuSK appears integral to the developing neuromuscular junction, its primary role in the adult is suspected to involve maintenance of structural integrity of the postsynaptic membrane [13, 14]. Weakness in MuSK-MG has been associated with decreased postsynaptic AChR density, decreased synaptic response to ACh release, and reduced presynaptic ACh release at high rates of activity [1, 10, 11]. Ultrastructural features of AChR-Ab+ MG include mild, nonspecific myopathic features with myofibrillar atrophy as the most prominent abnormality [15]. Histological studies of MuSK-MG patients revealed an increase in COX negative staining fibers when compared to AChR-Ab+ MG, suggestive of an underlying mitochondrial dysfunction [4, 16]. Mitochondrial abnormalities with marked myopathic features have also been described in MuSK-MG patient via electron microscopy [15]. Yet, no difference was observed in the amount of mtDNA mutations between AChR-Ab+ and MuSK Ab+ patient groups [4]. The significance of mitochondrial dysfunction in MuSK-MG is yet to be determined.

The presence of electrical myotonia with EDX testing has yet to be reported in patients with MuSK-MG. Electrical myotonia is a prolonged and spontaneous electrical discharge emanating from a single muscle fiber following stimulation or a needle puncture which characteristically waxes and wanes in both amplitude and frequency. Both clinical and electrical myotonia have been observed in diverse neuromuscular disorders, both inherited and acquired (reviewed in Table 1). Myotonic discharges result from chronically depolarized muscle membranes. This may be secondary to an ion channel dysfunction (including sodium, chloride, and potassium channels), associated with muscle and/or muscle membrane damage, or related to genetic mutations which lead to muscle membrane ion channel dysfunction [17, 18]. There is one existing report of a patient presenting with Ach-R+ MG and myotonia, which was independent of the MG diagnosis as it was associated with a novel sodium channel mutation [19].

At this point, any pathophysiological explanation for our observation of electrical myotonia in these two patients with MuSK-MG is conjectural. One possibility is that electrical myotonia is associated with mitochondrial dysfunction, reflected by increased number of mtDNA mutations and COX negative staining muscle fibers, which may possibly be associated with alterations in ion channel function. An isolated case report has described electrical myotonia in the setting of mitochondrial dysfunction related to chronic progressive external ophthalmoplegia (CPEO) [20]. One hypothesis proposed for this finding is that insufficient energy production in the setting of mitochondrial dysfunction may lead to a secondary channelopathy, resulting in myotonia [4, 20]. However, the muscle biopsy obtained in case one failed to reveal COX negative staining muscle fibers, other signs of mitochondrial dysfunction, or associated myopathy, and myotonia is generally not observed even in severe phenotypes associated with mitochondrial cytopathy. An alternative possibility is that an additional, coexisting pathogenic autoantibody is present which contributes to ion channel dysfunction. We feel that having observed two similar cases in a relatively short time period makes it unlikely that two independent processes (MuSK-MG and a disorder resulting in myotonia) were coincident.

The limitations of this case report include the lack of further extensive testing (e.g., short and long exercise test and genetic testing for inherited channelopathies) to exclude alternative causes of electrical myotonia. Yet given the absence of family history, the specificity of MuSK antibodies, and the significant response to immunomodulatory treatment, that possibility appears remote. Abolishment of electrical myotonia after immunotherapy may provide an additional argument to the autoimmune basis of the phenomenon, though repeat EDX testing was not available for our patients.

The presence of electrical myotonia in these two patients with MuSK-MG suggests that MuSK-MG may be considered in the differential diagnosis for disease processes which feature electrical myotonia. Such may significantly impact diagnostic workup, which might involve costs associated with both genetic testing and muscle biopsy. The fact that these observations were made in two separate patients in the same institution may suggest that this finding is more common than currently recognized. The finding of electrical myotonia in these two patients with MuSK-MG generates questions which will require further evaluation, as the need for further clarification of the mechanism of electrical myotonia in MuSK Ab MG is significant. Further defining of the cause of the myotonia would likely offer a better understanding of the pathophysiology of MuSK Ab in MG.

## 4. Conclusion

To our knowledge, this is the first case report to describe electrical myotonia in MuSK-MG. These findings call for inclusion of MuSK-MG in the differential diagnosis of myotonic disorders and potentially indicate an additional, alternative pathway for investigation of the pathophysiology of this disorder. Further studies are needed to better define the prevalence and cause of electrical myotonia in these patients and the possible role of mitochondrial dysfunction in MuSK-MG.

## Figures and Tables

**Table 1:** 

Differential diagnosis of electrical myotonia	
Myotonic dystrophies (1 and 2)	Severe subacute denervation
Myotonia congenita	Welander myopathy
Schwartz-Jampel syndrome	Myotubular myopathy
Paramyotonia congenita	Centronuclear myopathy
Hyperkalemic periodic paralysis	Hereditary vacuolar myopathy
Pompe disease/acid maltase def.	Branching enzyme deficiency
Polymyositis	Debranching enzyme deficiency
Cholesterol lowering agent myopathy	Chronic progressive external ophthalmoplegia
Colchicine myopathy	Monocarboxylic acid myotonia
Hypothyroidism	20,25-Diazacholesterol myotonia
